# Peer effects on adolescent smoking: Are popular teens more influential?

**DOI:** 10.1371/journal.pone.0189360

**Published:** 2018-07-12

**Authors:** Juan David Robalino, Michael Macy

**Affiliations:** 1 Department of Economics, Cornell University, Ithaca, NY, United States of America; 2 IZA – Institute for the Study of Labor, Bonn, Germany; 3 Department of Information Science, Cornell University, Ithaca, NY, United States of America; University of California, San Diego, UNITED STATES

## Abstract

Previous research on adolescent cigarette adoption has focused on peer influence and the perceived status gain from smoking but has ignored the status effects on peer influence. We analyze adolescent peer effects on cigarette consumption while considering the popularity of peers. The analysis is based on a four wave panel survey representative of American high school students. We measure peers’ popularity by their eigenvector centrality in high school social networks. Using lagged peers’ behavior, school fixed effects, and instrumental variables to control for homophily and contextual confounds, we find that the probability of smoking the following year increases with the mean popularity of smokers, while the popularity of non-smokers has the opposite effect. These effects persist seven and fourteen years later (wave 3 and 4 of the data). In addition, the probability of smoking increases with the smoking propensity of the 20% most popular teens and decreases with the smoking propensity of the bottom 80%. The results indicate the importance of knowing not only the smoking propensity within a school but also the location of smokers within the social hierarchy.

## Introduction

Identifying the drivers of smoking adoption among youth remains a public health priority. Initiating smoking at a young age is correlated with smoking more cigarettes per day and with a lower probability of quitting later in life [1, 2]. More than 480,000 deaths are attributed to cigarette smoking every year in the US alone. At the current rate of adoption, 5.6 million Americans under 18 –about 1 of every 13– will die early from a smoking-related illness.

Previous research points to peer influence as an important cause of adolescent smoking [3–9]. Adolescents are especially vulnerable to social influence as they try to fit in with their peers [10, 11]. Numerous studies suggest that some smokers believe that smoking promotes social status [12–15], and yet previous research has failed to test a key implication: that the propensity to smoke may increase with the popularity of smokers among peers. In other contexts, peer influence has been found to increase with their social status [16], but no previous studies have empirically tested the effects of the social status of adolescent smokers on the spread of smoking through peer networks. The need to empirically test this status-belief explanation is the starting point for our study.

## Previous research on popularity and smoking

Several studies have analyzed the link between popularity of adolescents (usually measured by the number of incoming friendship nominations) and their smoking behavior. For example, Valente et al. [12] and Ennett et al. [13] found a positive relationship between popularity and smoking; Alexander et al. [14] found that popular students are more likely to smoke in schools with high smoking rates, and less likely in schools with low smoking rates. Michell and Amos [15] used qualitative data from Scottish schoolgirls and found that popular girls smoke to maintain their image while some unpopular girls smoke with the hope of gaining social status, but no effect in the mid-popularity range.

These studies indicate that smoking may be used as a strategy to climb the social ladder, based on the belief that smoking will lead to an increase in social status. However, they do not test the peer effects of smokers’ social status. To address this gap, we reverse the causal arrow: instead of testing whether smoking affects status, we test how the popularity of smokers affects their influence on the behavior of their peers. We posit that the relative popularity of smokers and non-smokers will condition whether smoking will be associated with social status in that population. To the best of our knowledge, this is the first study of the effect of smokers’ popularity on peer influence for smoking.

It is worth noting that there have been anti-smoking field interventions that relied implicitly on the hypothesis that we are testing. For example, to promote an anti-smoking campaign in high schools, a field experiment explicitly recruited individuals nominated by fellow students as “influential” [17], and their intervention proved successful in comparison to the smoking rates of the control high schools that did not recruit any students. In short, this research assumed status differences in influence but did not test that assumption. Our study is motivated by the need to empirically test an assumption that informs intervention strategies not only into smoking but into other public health, advertising, and electoral campaigns.

## The reflection problem

Like most previous studies of peer influence on adolescent smoking, we rely on observational data. Seminal work by Manski [18] identifies the inherent difficulty in estimating peer effects with observational data, which he refers to as the reflection problem. According to Manski, a correlation between the average behavior of the group and an individual’s behavior can be attributed to three possible mechanisms:

- *endogenous effects*, where an individual’s choice is influenced by the choices of the group, as occurs with peer effects on behavior;
- *exogenous (contextual) effects*, where individuals in a given group may behave similarly because the whole group has experienced an (unobserved) exogenous shock;
- *correlated effects*, where individuals in a group behave similarly because they have similar unobservable characteristics and self-select into the group.

The challenge with observational data is how to disentangle endogenous effects from contextual and correlated effects. Several identification approaches have been used. Sacerdote [19] studied peer effects on the Grade Point Average (GPA) of college students using exogenous group formation from random allocation to college dorms in order to control for peers’ self-selection and homophily. He also used peers’ lagged GPA (from high-school) to control for contextual confounds.

In the absence of random allocation of peers, several studies of peer effects on smoking have used broad pseudo-exogenous peer groups, controls for school level fixed effects, as well as instrumental variables for peers’ behavior. Lundborg [6] used the smoking rate among classmates as peer referents; Fletcher [3] used the school grade; Clark and Lohéac [9] used lagged behavior at the grade level (i.e., the smoking rate during the previous year); Norton et al. [20], Gaviria and Raphael [5] and Powell et al. [4] used the whole school. To account for contextual cofounds, Gaviria and Raphael [5], and Powell et al. [4] controlled for cigarette prices and public policy variables, while Lundborg [6], Clark and Lohéac [9], and Fletcher [3] included school fixed effects. Norton et al. [20], Gaviria and Raphael [5], Powell et al. [4], and Fletcher [3] also instrumented the behavior of peers using household or neighborhood characteristics. All these studies found strong evidence of peer influence on cigarettes use.

In line with previous studies, we use the lagged behavior of peers at the school grade level to control for homophily and include school fixed effects to control for contextual confounds. Nevertheless, the observed effects from multivariate models remain susceptible to unobserved heterogeneity. We therefore also use instrumental variables to test the robustness of the causal inferences.

## Data and methods

**Fig 1:**
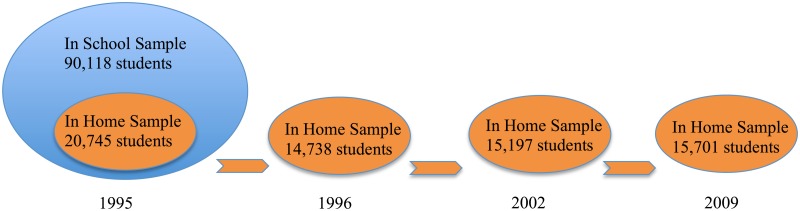
Data structure.

The present study is based on data from the National Longitudinal Survey of Adolescent Health (AddHealth), a four-wave panel (1995, 1996, 2002 and 2009) administered to a representative sample of American High Schools. This data set contains detailed information about substance use as well as rich social network data with which to measure the popularity of students. The multi-wave longitudinal data also includes identifiers for grade, year, school, and detailed measures of household and parents’ characteristics. The In-School survey conducted in 1995 covered 90,118 adolescents in 144 schools. A representative sub-sample of 20,745 students also completed the 1995 In-Home wave 1 survey which included more detailed information such as family income and parents’ smoking habits. Finally, about 15,000 students were surveyed again in 1996 (In-Home survey wave 2), as well as 2002 (In-Home wave 3) and 2009 (In-Home wave 4; see Fig 1). After excluding individuals with missing information and schools with insufficient social network data, we have a sample of about 66,000 peers and a core sample of about 7,500 individuals.

### Popularity measure

AddHealth identifies peers by asking each respondent to name their five closest male friends within the school and their five closest female friends in order of importance (i.e., best friend, second best friend, etc). These nominations form the social network of the school and can then be used to measure the popularity of each respondent by their *eigenvector centrality*. Intuitively, the popularity of individual *i* will be proportional to the popularity of peers who nominated *i* as a friend, hence being nominated by popular teens makes you more popular. Formally, the centrality *v*_*i*_ of individual *i* in school *s* is

$$\begin{array}{c} v_{i}=\frac{1}{\lambda }\sum_{j}v_{j}a_{ji} \end{array}$$

where *a*_*ij*_ = 1/*n*_*ij*_ if individual *i* nominated individual *j* as his/her *n*^*th*^ best friend and *a*_*ij*_ = 0 otherwise; or equivalently using vector notation we recover the eigenvector expression $\lambda v=A_{s}^{\prime }v$, where *A*_*s*_ is the so called adjacency matrix of the social network in school *s* and *λ* is the largest eigenvalue –in order to ensure that all the elements of *v* are positive as guaranteed by the Perron–Frobenius theorem. This weighted-eigenvector centrality (i.e., weighted by the inverse of the friendship nomination order) is preferred as it also takes account of the difference between being the best friend of many people and being the fifth best friend of many people. In the working paper [21] we obtain similar results using in-degree, out-degree, and Katz–Bonacich centrality.

We compare the centrality measure across school/grades in two ways: by standardizing the measure, and by classifying respondents into centrality quintiles for each grade level (from the 20% most popular to the 20% least popular).

Table 1 describes ‘popular’ teens by regressing centrality on respondent attributes. The results show that popular teens tend to be older, white, from higher income households, well groomed, play sports and they tend to be physically mature and attractive. In contrast, un-popular teens tend to be black or hispanic, overweight, foreign, and new to the school.

**Table 1:** Correlates of popularity. Weighted standardized eigenvector centrality.

Smoke—Tried 1995	0.033 (0.028)	Black	-0.367*** (0.046)	Candid (0/1)	0.003 (0.030)
Smoke—Everyday 1995	-0.022 (0.050)	Hispanic	-0.123*** (0.045)	Attractive personality (0/1)	-0.018 (0.038)
Cigs. available at home	-0.044 (0.030)	Asian	0.014 (0.068)	Well groomed (0/1)	0.060* (0.032)
Male	-0.002 (0.031)	Other	-0.13 (0.162)	Physically attractive (0/1)	0.182*** (0.037)
Age	0.298* (0.164)	Weekly earnings	-0.027 (0.018)	Physically mature (0/1)	0.118*** (0.032)
Age sq.	-0.009* (0.005)	Overweight (0/1)	-0.151*** (0.028)	Const.	-2.719* (1.390)
HH income	0.922*** (0.346)	Sports 1-2 times/week	0.055 (0.033)	Degrees of freedom	148
Foreign	-0.096** (0.039)	Sports 3-4 times/week	0.094** (0.040)	R^2^	0.154
New student	-0.152*** (0.033)	Sports 5+ times/week	0.198*** (0.039)	N	7169

OLS regression. Standard errors clustered at the school level are shown in parenthesis.

*Significance at the 10% level;

**Significance at the 5% level;

***Significance at the 1% level.

### Smoking measures

The items on smoking behavior differ slightly between the In-Home and In-School survey instruments. For the In-Home survey, we consider as regular smokers those who smoked *everyday* during the month prior to the interview. We also classify casual smokers as those who *tried* at least one cigarette in the 30 days prior to the interview. More precisely, for each wave we also measure the number of cigarettes consumed per month. In wave 4 (for which the interview took place fourteen years after wave 1) we also code respondents having *ever* smoked everyday for at least one month (thus, accounting for individuals who may have smoked only between interviews); the age at which individuals tried their first cigarette; and the age at which individuals started smoking everyday. The top panel of Table 2 summarizes these measures. In 1996, 32% of students tried cigarettes and 11% smoked everyday; by 2009, 21% were regular smokers. Our estimation strategy assumes that the correlations between smoking in 1995 and in subsequent years are not so high as to introduce multicollinearity. In S1 Table in the supporting information we present these correlations and the first column shows that there is considerable variation between smoking in 1995 and subsequent years (ranging from a correlation with smoking in 2009 of 0.3, to a correlation with “trying cigarettes in 1996” of 0.53).

**Table 2:** Summary statistics.

	Mean	SD	N
***Smoking***			
Tried by 1995	0.25	0.43	7620
Tried 1996	0.32	0.47	7620
Everyday 1996	0.11	0.31	7620
Everyday 2002	0.17	0.37	7620
Everyday 2009	0.21	0.41	6293
Everyday by 2009	0.43	0.49	6290
# cig./month 1996	157.01	238.05	1860
# cig./month 2002	302.8	260.08	1679
# cig./month 2009	274.74	281.78	2179
Age first cigarette	15.84	3.49	3955
Age smoked everyday	17.38	3.38	2661
***Peers in 1995***			
*Smoking rate among:*			
20% most popular	0.16	0.37	13394
80% least popular	0.16	0.37	52792
*Mean popularity of:*			
Smokers	-0.04	0.87	10657
Non-smokers	0.01	1.02	55529

Peer smokers are those who smoke at least “once or twice a week”. Popularity is defined by standardized weighted-eigenvector centrality.

For peers in the In-School survey, we define as smokers those who reported smoking “once or twice a week” to “daily”. We intentionally use a high threshold of consumption for peers because we suspect that influence arises from regular users who may influence an (initially) soft consumption to the newly initiated individuals, which may eventually result in regular smoking later on (yet, all results are similar when we measure peer’s smoking as having *tried* cigarettes). The bottom panel of Table 2 summarizes the smoking patterns of peers classified by their standardized weighted-eigenvector centrality. On average, there is no difference in the smoking propensity of the 20% most popular and the 80% least popular (both have a smoking propensity of 0.16). The standardized popularity of smokers and non-smokers is near zero, indicating that both of these groups have average popularity (see the working paper [21] for detailed summary statistics).

### Model specification

We test the effects of popularity on peer influence using three related models. With each model, we regress smoking in each of the last three panel waves on peers’ smoking in the first panel wave (the 1995 In-School survey) while controlling for school fixed effects.

Eq (1) models the probability of smoking as a probit regression of the smoking behavior $Y_{i,s,g}^{t}$ of individual *i* at time *t* on the mean popularity of smokers, ${\bar{P}}_{s,g,y=1}^{t_{0}}$, and the mean popularity of non-smokers, ${\bar{P}}_{s,g,y=0}^{t_{0}}$, controlling for the smoking propensity, ${\bar{Y}}_{s,g}^{t_{0}}$, at the grade level *g* in school *s* during the first wave, *t*_0_. Formally we estimate the following equation:

$$\begin{array}{c} Probit\left(Y_{i,s,g}^{t}=1|X_{i},s,g\right)=c+z_{s}+\beta X_{i}+\alpha_{1}{\bar{P}}_{s,g,y=0}^{t_{0}}+\alpha_{2}{\bar{P}}_{s,g,y=1}^{t_{0}}+\alpha_{3}{\bar{Y}}_{s,g}^{t_{0}} \end{array}$$ (1)

where *X*_*i*_ is a vector of demographics and household characteristics and *z*_*s*_ is a school fixed effect. Eq (1) formalizes the hypothesized peer effects as *α*_2_ > 0 > *α*_1_, that is, the greater the popularity of smokers, the more likely an individual is to smoke in the future; and the greater the popularity of non-smokers, the less likely an individual is to smoke in the future.

Eq (2) models the smoking propensity of the 20% most popular teens, ${\bar{Y}}_{s,g,Pop}^{t_{0}}$, and the smoking propensity of the 80% least popular teens, ${\bar{Y}}_{s,g,noPop}^{t_{0}}$, in school *s* and grade *g*:

$$\begin{array}{c} Probit\left(Y_{i,s,g}^{t}=1|X_{i},s,g\right)=c+z_{s}+\beta X_{i}+\alpha_{1}{\bar{Y}}_{s,g,Pop}^{t_{0}}+\alpha_{2}{\bar{Y}}_{s,g,noPop}^{t_{0}} \end{array}$$ (2)

Eq (2) formalizes the hypothesized peer effects as *α*_1_ > *α*_2_, representing a stronger influence from the popular teens.

Eq (3) models the smoking propensity ${\bar{Y}}_{s,g,q}^{t_{0}}$ of the teens in each popularity quintile *q* in school *s* and grade *g*:

$$\begin{array}{c} Probit\left(Y_{i,s,g}^{t}=1|X_{i},s,g\right)=c+z_{s}+X_{i}\beta +\sum_{q=1}^{q=5}\alpha_{q}{\bar{Y}}_{s,g,q}^{t_{0}} \end{array}$$ (3)

We hypothesize that *α*_5_ > *α*_*q*≠5_.

All three models are vulnerable to unobservable confounds, which we address using temporal lags and school fixed effects. Most teens go to their local public school and many parents have little flexibility to switch neighborhoods based only on school choices. Even when they consider the schools in their neighborhoods, they will presumably consider other aspects of the school, including academic performance, class sizes, and facilities, before considering the smoking propensity in the school. We control for new students and for parents who claimed to have chosen their neighborhood in part for the school quality, and we use lagged peers’ behavior to model the direction of influence. We also control for detailed tobacco policy in 1995 –excise tax, funds per capita for tobacco control, and marketing restriction on public transports and on billboards near schools. Most importantly, school fixed effects control for contextual factors such as the local price of cigarettes, the school’s rules about smoking, and the local sentiment towards smoking. Thus, our variation is among cohorts within schools, which minimizes the effects of self-selection into schools and contextual confounds.

### Instrumental variables

We use instrumental variables to provide additional support for the causal inferences based on temporal lags and school fixed-effects. For model 1 we instrument the popularity of smokers and non-smokers. We need instruments that determine the popularity of peers but do not influence the future smoking choices of others. Based on the correlates of popularity found in Table 1, we instrument peer smokers’ mean popularity using the percent of smokers who are new to the school, overweight, physically attractive, physically mature, well groomed, white, and foreign, as well as their mean household income and weekly earnings. We also instrument popularity among non-smokers using these same measures. These measures are only available for the In-Home sample; thus, we use the corresponding averages in the In-Home sample to instrument for the popularity of peers in the In-School sample (see Fig 1). Note that school fixed effects are also included in the first stage regression ensuring that instruments such as percentage of white students and mean household income are uncorrelated with the error term in the second stage regression. The percentage of physically attractive smokers should only affect the smoking choices of others through the resulting prestige and popularity of these smokers.

For model 2 we instrument the smoking propensity among popular and non-popular students. The instruments should affect the smoking choices of peers but not the smoking choices of other individuals. We use the percentage of parents who smoke, percentage of households with smokers, the percentage of parents who are home when school finishes, the percentage of black students, percentage of students with older siblings, the average household income and the average weekly earnings among the 20% most popular and, analogously, among the 80% least popular students. Peers’ parents smoking should only affect smoking choices of peers but not of other students.

## Results

Using peers’ lagged behavior, school fixed effects, and instrumental variables to control for contextual confounds and school selection, results for model 1 show that the probability that the respondent will take up smoking increases with a lagged measure of the mean popularity of peer smokers as measured by network centrality. Conversely, higher mean popularity of non-smokers decreases this probability. These patterns persist seven and fourteen years after peers’ behavior was measured (i.e., in waves 3 and 4 of the data). Similarly, by decomposing the smoking propensity of peers into the propensity of the 20% most popular teens and that of the 80% least popular, results for model 2 show that peer effects are mainly driven by the influence of the 20% most popular teenagers. Furthermore, in the long run we find a negative influence from the smoking propensity of the 80% least popular peers (in waves 3 and 4 of the data). Similar results apply to the number of cigarettes smoked per month as well as to the age of initiation (see S1 File for details). These patterns suggest that social influence on smoking among adolescents is conditioned by the social status of peers: individuals tend to follow the smoking behavior of popular peers and avoid the behavior of unpopular peers.

### Popularity of smokers and non-smokers

Table 3 summarizes results for model 1. Consistent with published results from previous studies of peer influence, the third row of the table shows an effect of the aggregate smoking propensity. In 1996, switching from a school grade where no one smokes to one where 25% of students smoke increases the probability of trying cigarettes by 4.8 percentage points and that of smoking every day by 4 percentage points, comparable to the effect of having access to cigarettes at home, and similar in magnitude to results reported in previous studies [3]. Yet, this peer effect vanishes by 2002 and 2009.

**Table 3:** Probability of smoking and popularity of smokers/non-smokers—Probit average marginal effects.

	Tried 1996	1996	2002	2009	by 2009
Mean popularity of smokers	0.045*** (0.006)	0.009 (0.009)	0.026*** (0.008)	0.026*** (0.007)	0.033*** (0.009)
Mean popularity of non-smokers	-0.059*** (0.015)	-0.013 (0.011)	-0.032*** (0.012)	-0.028* (0.016)	-0.059*** (0.018)
% smokers in grade	0.191* (0.099)	0.163** (0.071)	-0.053 (0.074)	-0.02 (0.100)	-0.116 (0.103)

Regressions include school fixed effects. Standard errors clustered at the school level are shown in parenthesis. Peer smokers are those who smoke at least “once or twice a week” in 1995. Peer variables are at the grade level. Includes all covariates from S2 Table in the supporting information.

*Significance at the 10% level;

**Significance at the 5% level;

***Significance at the 1% level.

Our main result is presented in the first two rows of Table 3: the mean popularity of smokers increases the probability of an individual smoking, and the mean popularity of non-smokers decreases this probability. In contrast to the aggregate peer effect, the effect of peers’ popularity persists in 2002 and in 2009, suggesting that in the long run the popularity of peer smokers is more important than how many peers smoked. The left panel suggests that an increase of a standard deviation in the mean popularity of smokers results in a increase of 4.5 percentage points in the probability of trying cigarettes in 1996, while the same increase in the mean popularity of non-smokers reduces this probability by 5.9 percentage points. The fourth panel suggests that an increase of a standard deviation in the popularity of smokers will result in an increase of 2.6 percentage points in the probability of smoking everyday in 2009, fourteen years after having interacted with those peers, and the same variation in the popularity of non-smokers will reduce this probability by 2.8 percentage points. Similarly, the fourth panel suggests that an increase of a standard deviation in the mean popularity of smokers results in an increase of 3.3 percentage points in the probability of ever smoking daily by 2009 and the same increase in the popularity of non-smokers results in a decrease of 5.9 percentage points in the probability of ever smoking.

Note that the probability of smoking *everyday* in 1996 is not significantly affected by the popularity of peers. It seems that influence from peers’ popularity only produced casual smoking at that time (i.e. during high school years). Yet, the smoking rate in the grade is significant in this regression. We note that in 1996 the average age in the sample was 16 years old, thus, most smokers could not yet buy cigarettes legally. This may explain why the aggregate smoking propensity was more important for regular smokers in this period as it may have facilitated the supply of cigarettes among peers. If we only consider the subsample of students who had access to cigarettes at home as a proxy of students with weaker supply constraints, the effect from the smoking rate has no statistical significance and we get a significant effect from the popularity of smokers (see S3 Table in the supporting information).

#### Robustness

One possible explanation for our results is that popular peers may not be more influential due to their social status, but may simply reach more people and that their influence is a simple one, just like that of other peers, but towards more friends. To test this, we include the smoking propensity of all nominated friends for each individual. S4 Table (in the supporting information) summarizes the results. The smoking propensity among direct friends absorbs the effect of the smoking propensity in the grade and it is still significant in 2002 and in 2009 (although there is potential self-selection). In contrast, the effects of the mean popularity of smokers and non-smokers remain essentially unchanged. This is consistent with the hypothesis that popular peers have a stronger influence than non-popular peers.

The persistence of peer effects may be due to addiction, which can be expected to induce serial correlation in smoking measures for latter periods. To control for serial correlation of smoking in the latter waves, we consider separately subsamples of smokers and non-smokers in early waves and predict their probability of starting/quitting smoking in latter waves. The results reported in S5 Table suggest that addiction may eventually override some peer effects in the long term, but not all. The first three columns indicate that individuals who were not regular smokers in 1996, and even those who had never been regular smokers by 2002, continue to be influenced by the popularity of high school smokers/non-smokers to adopt smoking by 2002 or 2009. The last two columns indicate that, among those who smoked in high school (1996), those who were exposed to popular smokers, compared to those who were not exposed, were still more likely to be smoking as late as 2002, but not after. And among those who smoked in 2002, we find no residual effect of the 1995 popularity of smokers by 2009. A reasonable conclusion is that by 2009, those who began smoking seven or more years earlier may indeed be hooked and their probability of quitting may be less susceptible to the popularity of smokers and non-smokers in high school.

S6 Table (in the supporting information) reports results of a robustness test using instrumental variables to infer causality. The results confirm those reported in Table 3, although, as expected, we loose some statistical significance. The instruments are highly significant in the first stage regression (J statistics between 13.2 and 22.2 corresponding to p-values of zero for the instruments’ joint significance in the first stage regressions); over-identification tests fail to reject the validity of our instruments and Wald test of exogeneity fail to reject the exogeneity of the popularity of smokers and non-smokers’ suggesting that a single equation probit model may be more appropriate (we can reject exogeneity at the 10% level in one regression, having ever smoked regularly by 2009; yet, results still hold).

### Smoking propensity of popular peers

S7 Table (in the supporting information) reports results estimated from Eq (2). The results suggest that peer influence from smokers is driven mainly by the 20% most popular peers. If all of the 20% most popular teens in the grade smoked, the probability of trying cigarettes the following year would increase by 17.8 percentage points (statistically significant at the.01 level), while if all of non-popular teens smoked, resulting in a much larger population of smokers, the increase in this probability would not be statistically different from zero. In fact, in 2002 and 2009, the bottom 80% seem to have a negative influence, that is, the more of these less-popular peers smoke, the less likely an individual is to pick up smoking later in life (although the effect is no longer statistically significant in 2009).

#### Robustness

S8 Table (in the supporting information) reports the results for the instrumental regression for Model 2. The results are qualitatively similar, but the peer influence of popular students is not statistically significant. While the model fails the over-identification test only in the 1996 measures, we cannot reject the null hypothesis that the smoking rates of smokers/non-smokers are exogenous, suggesting that a probit model may be more appropriate.

### Smoking propensity by popularity quintiles

S9 Table (in the supporting information) reports results for the effects estimated in Eq (3). The effects are similar to those in S7 Table, although the more granular cut of peer groups makes the pattern less striking. The top popularity quintile is the main driver of influence, and we find *negative* influence from the smoking rates of the least popular quintiles.

### Heterogeneity

We tested for possible interaction effects between the influence from smokers’ status and individual characteristics (gender, age, age relative to grade mean, physical attractiveness rated by interviewer, a dummy for new students, a dummy for foreigners and a dummy for parents claiming to have chosen the neighborhood partly for schools’ quality), as well as individual’s own network variables (the popularity of the respondent; a dummy signaling whether the respondent’s best friend also nominated him/her as a best friend; and the overall percentage of reciprocal ties from the individual’s nominations), and school social network characteristics (network density of the school). No statistically significant interaction effects were observed, suggesting that all students are vulnerable to the influence of popular smokers (see [21] for details.).

## Conclusion

There is a general consensus in the literature that adolescents are susceptible to peer influences, to engage in risky behaviors, including smoking. Previous studies have identified the desire for social status as an important motivation. The clear implication is that influence on smoking behavior from popular peers is stronger than influence from unpopular peers. We test this hypothesis using four waves of panel data from AddHealth. These data are unusual in providing a nearly complete social network of the school population, with which to measure popularity as the respondent’s eigenvector centrality in the network. An important limitation of these data is “the reflection problem.” We address challenges to causal inference posed by selection and contextual effects using school fixed effects, lagged measures of smoking adoption, and instrumental variables.

The results show that the probability of smoking increases with the lagged popularity of smokers, while the popularity of non-smokers has the opposite effect. These effects persist up to fourteen years after exposure to peers’ behavior. Drilling down, we find that most of the aggregate peer effects come from the smoking propensity of the 20% most popular peers. Importantly, we also find evidence of negative social influence, in which respondents avoid the behavior of unpopular teens. The smoking propensity of the 80% least popular peers has a negative influence on individuals’ smoking in the long run (seven and fourteen years later).

These findings underscore the importance of the popularity of peers in predicting their influence. Not only is it important to know the smoking propensity in schools but also where the smokers are located in the social hierarchy. For example, our results suggest that two schools where 20% of teens smoke may experience very different trajectories in the incidence of smoking: If those 20% who smoke are the most popular teens, the probability of smoking for students the next year can be expected to increase by 18 percentage points, while if they are among the least popular, the probability would not increase by a statistically significant amount. Collecting the entire social network of schools to compute popularity with a network centrality may be costly. Yet, Banerjee et al. [22] find that asking who are the influential people to a subsample of individuals can lead to accurate identification of central people.

These results point to several possible extensions. Although status has been shown to condition peer effects on the diffusion of a number of social contagions, more research is needed to know if popularity conditions peer influence on a range of other risky behaviors, including binge drinking, drug abuse, and stigmatized sexual behaviors. Equally important, we need to know if these status effects extend as well to positive adolescent behaviors such as academic achievement and sports.

## Supporting information

S1 Table Correlation across measures of smoking. (PDF)

S2 Table Probability of smoking—Probit average marginal effects. (PDF)

S3 Table Probability of smoking every day in 1996 for students with and without access to cigarettes at home—Probit average marginal effects. (PDF)

S4 Table Probability of smoking controlling for smoking of direct friends—Probit average marginal effects. (PDF)

S5 Table Subsamples of smokers/non-smokers to test for serial correlation in smoking measures—Probit average marginal effects. (PDF)

S6 Table Instrumental variable probit regression model 1: Popularity of smokers and non-smokers. (PDF)

S7 Table Probability of smoking and smoking rates among popular and non-popular students—Probit average marginal effects. (PDF)

S8 Table Instrumental variable probit regression model 2: Smoking rate among popular and non-popular. (PDF)

S9 Table Probability of smoking and smoking propensity by popularity quintiles of peers—Probit average marginal effects. (PDF)

S1 File Number of cigarettes smoked per month and age of initiation. (PDF)
