# Supplementary material for: Peer effects on adolescent smoking: Are popular teens more influential?
Source: PLoS One. 2018 Jul 12;13(7):e0189360. doi: 10.1371/journal.pone.0189360 (PMC6042691; doi:10.1371/journal.pone.0189360)
Supplement: S1 Table — (PDF) [file pone.0189360.s001.pdf]

S1 Table. Correlation across measures of smoking.

	Tried by 1995	Tried 1996	1996	2002	2009
Tried by 1995	1				
Tried 1996	0.535	1			
1996	0.492	0.523	1		
2002	0.349	0.391	0.427	1	
2009	0.305	0.338	0.367	0.572	1
by 2009	0.43	0.488	0.399	0.56	0.59
