# Supplementary material for: Peer effects on adolescent smoking: Are popular teens more influential?
Source: PLoS One. 2018 Jul 12;13(7):e0189360. doi: 10.1371/journal.pone.0189360 (PMC6042691; doi:10.1371/journal.pone.0189360)
Supplement: S2 Table — (PDF) [file pone.0189360.s002.pdf]

S2 Table. Probability of smoking – probit average marginal effects.

	Tried 1996	1996	2002	2009	by 2009
Mean popularity of smokers	0.045***	0.009	0.026***	0.026***	0.033***
	(0.006)	(0.009)	(0.008)	(0.007)	(0.009)
Mean popularity of non-smokers	-0.059***	-0.013	-0.032***	-0.028*	-0.059***
	(0.015)	(0.011)	(0.012)	(0.016)	(0.018)
% smokers in grade	0.191*	0.163**	-0.053	-0.02	-0.116
	(0.099)	(0.071)	(0.074)	(0.100)	(0.103)
Had tried smoking in 1995	0.383***	0.184***	0.152***	0.208***	0.390***
	(0.006)	(0.005)	(0.009)	(0.011)	(0.012)
Male	-0.002	0.000	0.034***	0.049***	0.071***
	(0.009)	(0.007)	(0.007)	(0.010)	(0.012)
Age (+ Age squ.)	0.176***	0.101**	0.078*	0.086	0.179***
	(0.062)	(0.045)	(0.045)	(0.062)	(0.069)
White	*Omitted*				
Black	-0.108***	-0.081***	-0.099***	-0.035*	-0.066***
	(0.015)	(0.010)	(0.013)	(0.019)	(0.022)
Hispanic	0.001	-0.018	-0.066***	-0.069***	-0.02
	(0.018)	(0.014)	(0.018)	(0.018)	(0.027)
Asian	-0.022	-0.030**	-0.025	-0.035	0.019
	(0.027)	(0.014)	(0.022)	(0.034)	(0.032)
Other	0.021	0.054	-0.082***	0.008	0.011
	(0.038)	(0.041)	(0.030)	(0.057)	(0.054)
Foreign	-0.023	-0.019	-0.005	-0.028	-0.041*
	(0.025)	(0.024)	(0.021)	(0.026)	(0.022)
Out of school in 1996	0.062***	0.063***	0.066***	0.057**	0.076***
	(0.023)	(0.013)	(0.018)	(0.024)	(0.028)
New student in 1995	0.004	0.004	-0.009	0.020*	0.024
	(0.011)	(0.007)	(0.011)	(0.012)	(0.016)
Weekly earnings ($100)	0.01	0.011***	0.002	0.006	0.015*
	(0.007)	(0.004)	(0.004)	(0.006)	(0.008)
Household Income ($1000 000)	-0.057	-0.231*	-0.286*	-0.237	-0.316**
	(0.119)	(0.134)	(0.148)	(0.203)	(0.145)
Moved partly for school quality	-0.01	-0.013*	-0.014	-0.017	-0.012
	(0.010)	(0.007)	(0.008)	(0.011)	(0.012)
Mother smokes	0.007	0.028***	0.014	0.030***	0.033**
	(0.012)	(0.008)	(0.011)	(0.011)	(0.015)
Father smokes	0.023**	0.016**	0.035***	0.014	0.009
	(0.011)	(0.006)	(0.009)	(0.010)	(0.012)
Cigarettes at home	0.046***	0.043***	0.033***	0.055***	0.069***
	(0.012)	(0.007)	(0.009)	(0.011)	(0.015)
Excise tax per cigarette pack	-0.012***	-0.012	0.005	0.000	-0.002
	(0.003)	(0.008)	(0.004)	(0.003)	(0.005)
Tobacco policy funds per capita	0.666***	1.730*	1.566**	1.028***	2.482***
	(0.240)	(1.012)	(0.700)	(0.391)	(0.668)
Marketing prohibited near schools	0.01	-0.027	0.225	0.098	0.094
	(0.126)	(0.118)	(0.157)	(0.147)	(0.167)
Marketing restricted on public transport	-0.996***	-1.466	-0.566	-0.151	-2.552***
	(0.186)	(1.077)	(0.670)	(0.401)	(0.668)
Pseudo R sq.	0.258	0.363	0.153	0.154	0.193
N	7611	7151	7580	6243	6292

Regressions include school fixed effects. Standard errors clustered at the school level are shown in parenthesis. Peer smokers are those who smoke at least “once or twice a week” in 1995. *Significance at the 10% level; **Significance at the 5% level; ***Significance at the 1% level.
