# Supplementary material for: Peer effects on adolescent smoking: Are popular teens more influential?
Source: PLoS One. 2018 Jul 12;13(7):e0189360. doi: 10.1371/journal.pone.0189360 (PMC6042691; doi:10.1371/journal.pone.0189360)
Supplement: S3 Table — (PDF) [file pone.0189360.s003.pdf]

S3 Table. Probability of smoking every day in 1996 for students with and without access to cigarettes at home – probit average marginal effects.

	Access to cigarettes at home	
	Yes	No
Mean popularity of smokers	0.035**	0.002
	(0.015)	(0.008)
Mean popularity of non-smokers	-0.015	-0.023**
	(0.019)	(0.011)
% smokers	0.102	0.210***
	(0.126)	(0.075)
N	2092	4625

Regressions include school fixed effects. Standard errors clustered at the school level are shown in parenthesis. Peer smokers are those who smoke at least “once or twice a week” in 1995. Peer variables are at the grade level. Includes all covariates from S2 Table. *Significance at the 10% level; **Significance at the 5% level; ***Significance at the 1% level.
