# Supplementary material for: Peer effects on adolescent smoking: Are popular teens more influential?
Source: PLoS One. 2018 Jul 12;13(7):e0189360. doi: 10.1371/journal.pone.0189360 (PMC6042691; doi:10.1371/journal.pone.0189360)
Supplement: S4 Table — (PDF) [file pone.0189360.s004.pdf]

S4 Table. Probability of smoking controlling for smoking of direct friends – probit average marginal effects.

	Tried 1996	1996	2002	2009	by 2009
Mean popularity of smokers	0.052***	0.007	0.028***	0.024***	0.037***
	(0.007)	(0.009)	(0.008)	(0.007)	(0.009)
Mean popularity of non-smokers	-0.065***	-0.012	-0.031**	-0.026	-0.053***
	(0.016)	(0.009)	(0.014)	(0.019)	(0.019)
% Smokers	0.117	0.087	-0.156**	-0.103	-0.237**
	(0.104)	(0.069)	(0.077)	(0.102)	(0.107)
% Direct friends smoking	0.117***	0.096***	0.113***	0.128***	0.172***
	(0.024)	(0.011)	(0.020)	(0.026)	(0.036)

Regressions include school fixed effects. Standard errors clustered at the school level are shown in parenthesis. Peer smokers are those who smoke at least "once or twice a week" in 1995. Peer variables are at the grade level. Includes all covariates from S2 Table. *Significance at the 10% level; **Significance at the 5% level; ***Significance at the 1% level.
