# Supplementary material for: Peer effects on adolescent smoking: Are popular teens more influential?
Source: PLoS One. 2018 Jul 12;13(7):e0189360. doi: 10.1371/journal.pone.0189360 (PMC6042691; doi:10.1371/journal.pone.0189360)
Supplement: S5 Table — (PDF) [file pone.0189360.s005.pdf]

S5 Table. Subsamples of smokers/non-smokers to test for serial correlation in smoking measures – probit average marginal effects.

Subsample – regular smoker in 1996:	No			Yes	
	2002	2009	by 2009	2002	2009
Mean popularity of smokers	0.015**	0.019**	0.031***	0.288***	0.049
	(0.007)	(0.009)	(0.011)	(0.085)	(0.106)
Mean popularity of non-smokers	-0.011	-0.028*	-0.056***	-0.274***	0.014
	(0.011)	(0.016)	(0.019)	(0.076)	(0.074)
N	6699	5583	5628	759	599

Subsample – regular smoker *by* 2002:	No		Yes
	2009	by 2009	2009
Mean popularity of smokers	0.017	0.001	0.031*
	(0.018)	(0.016)	(0.017)
Mean popularity of non-smokers	-0.040**	-0.049**	-0.006
	(0.018)	(0.021)	(0.029)
N	2550	3215	2033

Regressions include school fixed effects. Standard errors clustered at the school level are shown in parenthesis. Peer smokers are those who smoke at least “once or twice a week” in 1995. Peer variables are at the grade level. Includes all covariates from S2 Table. *Significance at the 10% level; **Significance at the 5% level; ***Significance at the 1% level.
