# Supplementary material for: Peer effects on adolescent smoking: Are popular teens more influential?
Source: PLoS One. 2018 Jul 12;13(7):e0189360. doi: 10.1371/journal.pone.0189360 (PMC6042691; doi:10.1371/journal.pone.0189360)
Supplement: S6 Table — (PDF) [file pone.0189360.s006.pdf]

S6 Table. Instrumental variable probit regression model 1: popularity of smokers and non-smokers.

	Tried 1996	1996	2002	2009	by 2009
Probit Coef.					
Mean popularity of smokers	0.145	0.314	0.578***	0.123	0.343*
	(0.198)	(0.298)	(0.224)	(0.204)	(0.202)
Mean popularity of non-smokers	-0.570**	-0.345	-0.349	-0.282	-0.640**
	(0.245)	(0.314)	(0.263)	(0.291)	(0.263)
Marginal effect					
Mean popularity of smokers	0.038	0.04	0.123	0.03	0.105
Mean popularity of non-smokers	-0.148	-0.044	-0.075	-0.069	-0.197
p-value Wald exogeneity test	0.282	0.286	0.11	0.731	0.082
J statistic	13.213	22.279	19.362	15.227	19.399
p-value J statistic (over-identification)	0.657	0.134	0.25	0.508	0.249
F statistic (Mean pop. smokers)	40.937	40.937	41.19	34.869	34.43
p-value F statistic (Mean pop. smokers)	0	0	0	0	0
F statistic (Mean pop. non-smokers)	30.453	30.453	30.332	22.887	23.158
p-value F statistic (Mean pop. non-smokers)	0	0	0	0	0
N	7073	6654	6996	5788	5830

Instrumental variables: percentage of white, foreigners, new students, overweight students, physically attractive, physically mature, well groomed, mean household income, and weekly earnings. IVs are computed both among smokers and among non-smokers. These variables are only available in the InHome survey, hence, we compute them from the InHome sample to instrument the mean popularity in the InSchool sample (see Fig 1). Regressions include school fixed effects. Robust standard errors are in parenthesis. Peer smokers are those who smoke at least "once or twice a week" in 1995. Peer variables are at the grade level. Includes all covariates from S2 Table. *Significance at the 10% level; **Significance at the 5% level; ***Significance at the 1% level.
