# Supplementary material for: Peer effects on adolescent smoking: Are popular teens more influential?
Source: PLoS One. 2018 Jul 12;13(7):e0189360. doi: 10.1371/journal.pone.0189360 (PMC6042691; doi:10.1371/journal.pone.0189360)
Supplement: S7 Table — (PDF) [file pone.0189360.s007.pdf]

S7 Table. Probability of smoking and smoking rates among popular and non-popular students – probit average marginal effects.

	Tried 1996	1996	2002	2009	by 2009
Smoking propensity:					
20% most popular	0.178***	0.055	0.170***	0.022	0.152**
	(0.056)	(0.040)	(0.037)	(0.063)	(0.067)
80% least popular	0.04	0.107**	-0.221***	-0.045	-0.330***
	(0.092)	(0.052)	(0.066)	(0.095)	(0.098)

Regressions include school fixed effects. Standard errors clustered at the school level are shown in parenthesis. Peer smokers are those who smoke at least "once or twice a week" in 1995. Peer variables are at the grade level. Includes all covariates from S2 Table. *Significance at the 10% level; **Significance at the 5% level; ***Significance at the 1% level.
