# Supplementary material for: Peer effects on adolescent smoking: Are popular teens more influential?
Source: PLoS One. 2018 Jul 12;13(7):e0189360. doi: 10.1371/journal.pone.0189360 (PMC6042691; doi:10.1371/journal.pone.0189360)
Supplement: S8 Table — (PDF) [file pone.0189360.s008.pdf]

S8 Table. Instrumental variable probit regression model 2: smoking rate among popular and non-popular.

	Tried 1996	1996	2002	2009	by 2009
Probit Coef.					
smoking rate among 20% most pop.	0.879	0.532	0.664	1.258	0.753
	(0.834)	(1.093)	(0.911)	(0.881)	(0.799)
smoking rate among 80% lest pop.	-0.284	0.021	-0.837	0.051	0.083
	(0.904)	(1.145)	(0.952)	(1.014)	(0.923)
Marginal effect					
smoking rate among 20% most pop.	0.228	0.066	0.141	0.305	0.235
smoking rate among 80% lest pop.	-0.074	0.003	-0.177	0.012	0.026
p-value Wald exogeneity test	0.869	0.667	0.964	0.236	0.26
J statistic	26.762	23.349	16.108	12.045	17.173
p-value J statistic (over-identification)	0.021	0.055	0.307	0.603	0.247
F statistic (sm. rate 20% most pop.)	35.284	35.284	35.033	32.804	32.794
p-value F statistic (sm. rate 20% most pop.)	0	0	0	0	0
F statistic (sm. rate 80% least pop.)	105.28	105.28	104.445	89.464	89.433
p-value F statistic (sm. rate 80% least pop.)	0	0	0	0	0
N	7769	7232	7736	6387	6433

Instrumental variables: percentage of black students, students with older siblings, parents who smoke, households with smokers, parents home after school finishes, average household income and average weekly earnings. IVs are computed both among the 20% most popular and the 80% least popular students. These variables are only available in the InHome survey, hence, we compute them from the InHome sample to instrument the mean popularity in the InSchool sample (see Fig 1). Regressions include school fixed effects. Robust standard errors are in parenthesis. Peer smokers are those who smoke at least “once or twice a week” in 1995. Peer variables are at the grade level. Includes all covariates from S2 Table. *Significance at the 10% level; **Significance at the 5% level; ***Significance at the 1% level.
