# Supplementary material for: Peer effects on adolescent smoking: Are popular teens more influential?
Source: PLoS One. 2018 Jul 12;13(7):e0189360. doi: 10.1371/journal.pone.0189360 (PMC6042691; doi:10.1371/journal.pone.0189360)
Supplement: S9 Table — (PDF) [file pone.0189360.s009.pdf]

S9 Table. Probability of smoking and smoking propensity by popularity quintiles of peers – probit average marginal effects.

	Tried 1996	1996	2002	2009	by 2009
Smoking propensity:					
Top pop. quintile	0.161***	0.051	0.179***	0.02	0.144**
	(0.057)	(0.040)	(0.039)	(0.064)	(0.067)
2nd pop. quintile	0.072	0.038	-0.092	-0.024	-0.077
	(0.058)	(0.038)	(0.058)	(0.051)	(0.071)
3rd pop. quintile	0.078	0.064	-0.024	0.102*	0.067
	(0.051)	(0.051)	(0.057)	(0.057)	(0.070)
4th pop. quintile	-0.094*	-0.006	-0.026	-0.062	-0.242***
	(0.050)	(0.034)	(0.050)	(0.055)	(0.052)
5th pop. quintile	-0.018	0.008	-0.074*	-0.033	-0.053
	(0.053)	(0.030)	(0.045)	(0.058)	(0.069)

Regressions include school fixed effects. Standard errors clustered at the school level are shown in parenthesis. Peer smokers are those who smoke at least "once or twice a week" in 1995. Peer variables are at the grade level. Includes all covariates from S2 Table. *Significance at the 10% level; **Significance at the 5% level; ***Significance at the 1% level.
