# Supplementary material for: Peer effects on adolescent smoking: Are popular teens more influential?
Source: PLoS One. 2018 Jul 12;13(7):e0189360. doi: 10.1371/journal.pone.0189360 (PMC6042691; doi:10.1371/journal.pone.0189360)
Supplement: S1 File — (PDF) [file pone.0189360.s010.pdf]

S1 File. Number of cigarettes per month and age of initiation

In Table A we regress the number of cigarettes consumed per month, as well as the age at which individuals tried their first cigarette and the age at which they smoked everyday for the first time. To account for non-smokers, we estimate a Tobit censured equation analogous to equation (1). We find that the same patterns reported above regarding peers' popularity apply to the number of cigarettes and the age of initiation. Conditional on smoking, a standard deviation increase in the mean popularity of smokers increases consumption by 7 cigarettes per month in 1996, 50 cigarettes per month in 2002 and 8 per month in 2009; and the same variation in the popularity of non-smokers decreases consumption by 8 cigarettes per month in 1996, 80 cigarettes per month in 2002 and 20 in 2009.

Table A. Number of cigarettes smoked per month and age of initiation – Model 1 Tobit average marginal effects.

	Number of cigarettes/ month			Age	
	1996	2002	2009	1st cig.	smoked everyday
Mean popularity smokers	7.067***	50.330***	8.235**	-0.822***	-0.641***
	(1.849)	(2.457)	(3.361)	(0.227)	(0.158)
Mean popularity non-smokers	-8.142**	-79.163***	-19.214***	1.061***	1.101***
	(3.465)	(3.037)	(7.193)	(0.387)	(0.309)
% smokers	87.190***	89.301***	-64.441*	1.472	2.631
	(25.474)	(15.055)	(37.196)	(2.724)	(2.023)

Regressions include school fixed effects. Standard errors clustered at the school level are shown in parenthesis. Peer smokers are those who smoke at least "once or twice a week" in 1995. Peer variables are at the grade level. Number of cigarettes is computed by multiplying the answers to the questions "During the past 30 days, on how many days did you smoke cigarettes?" and "During the past 30 days, on the days you smoked, how many cigarettes did you smoke each day?." Includes all covariates from S2 Table.
*Significance at the 10% level; **Significance at the 5% level; ***Significance at the 1% level.

Regarding the age of initiation, conditional on ever trying cigarettes, we find that a standard deviation above the mean popularity of smokers advances the age at which individuals try their first cigarette by 10 months; and the same variation in the popularity of non-smokers delays the age at which individuals try their first cigarette by over a year. Similarly, conditional on ever smoking everyday, we find that a standard deviation above the mean popularity of smokers advances the age at which individuals started smoking everyday by almost 8 months; and the same variation in the popularity of non-smokers delays the age at which individuals started smoking everyday by over a year.

Table B reports estimates for Tobit regressions analogous to model (2). If all of the 20% most popular students smoked, cigarettes consumption would increase by 26 cigarettes in 1996, and by 334 cigarettes in 2002. The same variation advances the age of the first cigarette by almost three years and the age when an individual first started

Table B. Number of cigarettes smoked per month and age of initiation – Model 2 Tobit average marginal effects conditional on smoking.

	Number of cigarettes/ month			Age	
	1996	2002	2009	1st cig.	smoked everyday
Smoking propensity:					
20% most popular	25.972**	334.335***	-1.993	-2.829**	-2.461**
	(11.446)	(23.399)	(26.049)	(1.252)	(1.242)
80% least popular	60.358***	-119.426***	-64.777*	3.569	5.843***
	(21.144)	(5.754)	(35.779)	(2.478)	(1.785)

Regressions include school fixed effects. Standard errors clustered at the school level are shown in parenthesis. Peer smokers are those who smoke at least "once or twice a week" in 1995. Peer variables are at the grade level. Number of cigarettes is computed by multiplying the answers to the questions "During the past 30 days, on how many days did you smoke cigarettes?" and "During the past 30 days, on the days you smoked, how many cigarettes did you smoke each day?." Includes all covariates from S2 Table.
*Significance at the 10% level; **Significance at the 5% level; ***Significance at the 1% level.

smoking everyday by 2.5 years. If 25% of the 80% least popular teens (an equivalent 20% of the total students) cigarettes consumption would increase by 14 cigarettes in 1996, but it would decrease by 25 cigarettes in 2002. The same variation delays the age when first started smoking everyday by almost 1.5 years.[1]

These patterns are of particular importance in light of the existing evidence that smoking at a younger age has a strong impact in the number of cigarettes smoked and the probability of quitting smoking later in life (Everett et al., 1999; Lando et al., 1999). Popularity of peer smokers during the teenage years seems to be an important driver of these findings.
